# Dual therapy with allicin and metformin provides superior cardioprotection against doxorubicin-induced cardiotoxicity in rats compared to monotherapy

**DOI:** 10.3389/fphar.2025.1725943

**Published:** 2026-01-09

**Authors:** Mohammed Mojamel, Hassan Al-Mahbashi, Afif Al-Nabehi

**Affiliations:** 1 Department of Pharmacology &Therapeutics, Faculty of Medicine, Sana’a University, Sana’a, Yemen; 2 Department of Forensic Medicine and Clinical Toxicology, Faculty of Medicine, Sana’a University, Sana’a, Yemen; 3 Department of Medicine, Faculty of Medicine, Sana’a University, Sana’a, Yemen

**Keywords:** allicin, metformin, doxorubicin, cardiotoxicity, cardioprotection, rats, oxidative stress, antioxidant

## Abstract

**Background:**

Doxorubicin is a widely used chemotherapeutic agent; however, its clinical utility is limited by dose-dependent cardiotoxicity. Existing cardioprotective strategies are insufficient, showing that there is a need for safer and more effective alternatives.

**Objectives:**

This study evaluated the cardioprotective effects of metformin and allicin, individually and in combination, against doxorubicin-induced cardiotoxicity in rats.

**Methods:**

Fifty adult male Wistar albino rats were randomized into five groups (n = 10 each): The control group was administered normal saline (2 mL/kg, intraperitoneally, on days 7, 14, and 21); the DOX-only group received doxorubicin (6 mg/kg, intraperitoneally, on days 7, 14, and 21; cumulative dose 18 mg/kg); the DOX + Allicin group was given allicin (40 mg/kg/day, orally), the DOX + Metformin group received metformin (300 mg/kg/day, orally), and the DOX + Allicin+ Metformin group received both agents at these doses. Treatments were given orally once daily for 21 days. On day 22, blood samples and cardiac tissues were collected for biochemical and histopathological evaluation. Parameters assessed included body and heart weights, serum cardiac biomarkers (CK-MB, LDH, cTn I), antioxidant defenses (GSH, CAT, GPx, SOD), and oxidative stress indices (MDA, NO).

**Results:**

Both allicin and metformin significantly attenuated DOX-induced elevation of cardiac enzymes, with greater protection observed under combined therapy. Antioxidant markers (GSH, GPx, SOD, CAT, NO) increased significantly, whereas MDA levels decreased. Dual treatment produced superior effects compared to either agent alone, a finding further supported by marked histopathological improvement in cardiac tissues.

**Conclusion:**

Metformin and allicin each conferred significant cardioprotection against doxorubicin-induced cardiotoxicity, evidenced by the restoration of cardiac enzymes, reduction of oxidative stress, and improvement in myocardial histoarchitecture. Notably, combined therapy produced greater biochemical and structural recovery than either monotherapy, highlighting its enhanced overall cardioprotective efficacy.

## Introduction

Doxorubicin (DOX) is an anthracycline antibiotic derived from *Streptomyces peucetius* and serves as a cornerstone in the treatment of various malignancies, including breast cancer, leukemias, lymphomas, and sarcomas (Sinha et al., 2025). Its potent anticancer activity is mediated through multiple mechanisms, including DNA intercalation, inhibition of topoisomerase II, and the generation of reactive oxygen species (ROS), which lead to the apoptosis of malignant cells (Kciuk et al., 2023). Despite its broad clinical utility, the therapeutic potential of DOX is significantly limited by cumulative, dose-dependent cardiotoxicity, which may present as acute, early-onset, or delayed cardiomyopathy (Devi et al., 2025).

The mechanisms underlying DOX-induced cardiotoxicity (DOX-IC) are multifactorial, involving excessive ROS production, mitochondrial dysfunction, iron overload, and the activation of apoptotic and inflammatory pathways (Vitale, Marzocco and Popolo, 2024). These overlapping processes ultimately result in cardiomyocyte loss, ventricular remodeling, and impaired cardiac function (Thorn et al., 2011). Although various strategies—such as liposomal DOX formulations, dexrazoxane, dose fractionation, and antioxidant supplementation—have been investigated, they often provide only partial protection and may be constrained by high costs, additional toxicity, or reduced anticancer efficacy (Purgatorio et al., 2024). This highlights the urgent need for safe, affordable, and effective cardioprotective agents that can be co-administered without compromising the antitumor activity of DOX (Ibrahim et al., 2024).

Natural compounds with strong antioxidant and anti-inflammatory properties represent a promising therapeutic approach. Allicin, the principal bioactive component of garlic, exhibits potent antioxidant activity by enhancing the body’s endogenous defense systems and reducing oxidative damage (Deng et al., 2024). Its favorable safety profile and minimal systemic toxicity make it a suitable candidate as an adjuvant in chemotherapy regimens (Bala et al., 2024). Similarly, metformin, a widely prescribed oral antihyperglycemic agent for type 2 diabetes, has gained attention for pleiotropic effects beyond glucose regulation. Evidence indicates that metformin exerts antioxidant, anti-inflammatory, anti-apoptotic, and cardioprotective actions, particularly in contexts where mitochondrial dysfunction and oxidative stress drive cardiac injury (Foretz et al., 2023; Satyam et al., 2023). Mechanistically, metformin and allicin exert complementary—but non-redundant—cardioprotective actions as established across multiple preclinical studies. Metformin has been reported to activate AMPK, enhance mitochondrial bioenergetics, stimulate endothelial nitric oxide (NO) synthesis via eNOS phosphorylation, and augment Nrf2-driven antioxidant defenses. Allicin, in contrast, provides rapid thiol-dependent scavenging of ROS, inhibits lipid peroxidation, activates the Nrf2/Keap1 antioxidant pathway, and suppresses NF-κB–mediated inflammatory signaling. These literature-based mechanisms are referenced here solely to provide biological context for the rationale behind combining both agents; they were not directly measured in the present study. Importantly, although the mechanistic profiles of metformin and allicin have been individually characterized, no prior study has evaluated their combined effects within a unified DOX-induced cardiotoxicity model, leaving a critical evidence gap regarding whether their distinct pharmacodynamic signatures might yield additive or complementary cardioprotection.

Despite the extensive literature on garlic derivatives and the growing interest in repurposing metformin for cardiovascular protection, the dual administration of metformin and allicin has not been systematically investigated in DOX-IC.

Therefore, this study was designed to evaluate the individual and combined cardioprotective efficacy of metformin and allicin in a rat model of DOX-induced cardiotoxicity. By assessing serum biomarkers, oxidative stress indices, and histopathological changes, we aimed to determine whether dual therapy provides superior cardioprotection compared with monotherapy and to evaluate whether the observed biochemical and histological improvements are consistent with the potential complementary pharmacological actions reported for both agents.

## Materials and methods

### Drugs and chemicals

Doxorubicin (GLS Pharma Ltd., India), metformin (Denk Pharma GmbH & Co. KG, Germany), and allicin (Now Foods, United States) were used. All drugs were obtained from GMP-certified manufacturers. Commercial diagnostic kits for CK-MB, LDH, and cTn-I were obtained from Monlab (Spain), Labtest Diagnostica (Brazil), and Snibe Diagnostics (China), respectively. Antioxidant and oxidative stress assay kits (GSH, GPx, SOD, CAT, MDA, and NO) were obtained from Bio-Diagnostic (Egypt). All reagents were of analytical grade.

### Animals

Fifty adults male Wistar albino rats weighing between 150 ± 30 g were purchased from Sana’a University’s Animal House, Department of Biology. The rats were kept in polypropylene cages under standard laboratory conditions (temperature 25 °C ± 2 °C, 12-h light/dark cycle) with unrestricted access to standard pellet diet and water. Before the start of the experiment, the animals were allowed to acclimate for 1 week.

### Study design

The study was conducted over a period of 21 days, during which rats were randomly allocated into five groups (n = 10 per group) as follows: The control group received normal saline (2 mL/kg, i.p.) on days 7, 14, and 21; the DOX group received doxorubicin (6 mg/kg, i.p.) on days 7, 14, and 21, with a cumulative dose of 18 mg/kg (Octavia et al., 2012); the DOX + Allicin group received allicin (40 mg/kg/day, p.o.) for 21 days along with DOX as above; the DOX + Metformin group received metformin (300 mg/kg/day, p.o.) (Ma et al., 2018) for 21 days in addition to DOX as above; and the DOX + Metformin + Allicin group received both metformin (300 mg/kg/day, p.o.) and allicin (40 mg/kg/day, p.o.) (El-Sheakh et al., 2016) for 21 days with DOX as above. All oral treatments were freshly prepared in saline and administered by oral gavage, with doses adjusted daily according to body weight.

### Sample collection

On day 22, rats were fasted overnight and anesthetized with diethyl ether via inhalation in a closed glass chamber pre-saturated with approximately 2% vapor concentration. This concentration range (1.9%–3%) is well documented to induce a surgical plane of anesthesia in rodents (Flecknell, 2009; Underwood and Anthony, 2020). Adequate anesthesia was confirmed by the absence of the pedal withdrawal reflex. Following confirmation of deep anesthesia, euthanasia was performed by exsanguination via cardiac puncture, in accordance with the American Veterinary Medical Association (AVMA) Guidelines for the Euthanasia of Animals (2020), which recognize exsanguination as an acceptable secondary method of euthanasia following adequate anesthesia. This method has been widely applied in published experimental models in rats (Yesilyurt et al., 2019; Zhu et al., 2020).

Blood samples were collected by cardiac puncture, allowed to clot, and centrifuged at 3,000 rpm for 15 min to obtain serum (Centurion Scientific, model:K2002, United Kingdom), which was stored at −20 °C until analysis. Hearts were excised, rinsed with ice-cold phosphate-buffered saline (PBS, pH 7.4), blotted dry, and weighed. Each heart was divided into two parts: one fixed in 10% neutral-buffered formalin for histopathology (Bancroft and Gamble, 2008) and the other stored at −80 °C for biochemical assays (Ohkawa, 1978).

### Body weight

The alteration in body weight was measured before, during the experiment and at the end of the animal experiment.

### Relative heart weight

At the end of the study, the heart of each rat was weighed, and the relative heart weight (RHW) was calculated using the following equation:

$$\text{RHW }\left(\%\right)=\left(\text{Heart weight }/\text{ Body weight}\right)\times 100$$

### Biochemical analyses

#### Serum cardiac biomarkers

Serum levels of CK-MB (Anti and Líquido, n.d.) and LDH (Labtest Diagnostica, 2021) were measured spectrophotometrically, and cTn-I (Snibe, 2020) by chemiluminescence (MAGLUMI X3, Snibe Diagnostics). They were quantified using commercial diagnostic kits according to the manufacturers’ protocol.

#### Oxidative stress and antioxidant parameters

A tissue sample of 0.1 g was homogenized in 1 mL of phosphate-buffered saline (PBS) at PH 7.7 using an omni homogenizer (United States). The homogenate was then centrifuged according to the speed specified in the assay protocol to separate the supernatant. The collected supernatant was used for determination of oxidative stress and antioxidant parameters using standard colorimetric kits (Bio-Diagnostic, Egypt): MDA (Ohkawa et al., 1978), NO levels were assessed as total nitrite/nitrate (NOx) by the Griess reaction (Montgomery, 1971), GSH (Beutler, Duron and Kelly, 1963), GPx (Paglia and Valentine, 1967), Total SOD activity was measured using the method of Nishikimi et al. (1972), Nishikimi et al. (1972), and CAT (Aebi, 1984).

#### Histopathological examination

Formalin-fixed cardiac tissues were processed following the standard paraffin-embedding procedure according to established histological methods (Bancroft and Gamble, 2008; Wehrle et al., 2024). Briefly, cardiac samples were fixed in 10% neutral-buffered formalin, dehydrated through graded ethanol solutions, cleared in xylene, and embedded in paraffin wax. Sections of 4–5 μm thickness were cut and stained with hematoxylin and eosin (H&E) as described by Bancroft and Gamble (2008). The stained slides were examined under a light microscope at ×400 magnification (Olympus BX51, Japan) for histopathological alterations, including myofibrillar disarray, cytoplasmic vacuolization, necrosis, and inflammatory cell infiltration. A semi-quantitative scoring system (0–3) was applied to objectively evaluate myocardial injury. Each section was examined for myofiber degeneration or loss, cytoplasmic vacuolization, inflammatory cell infiltration, focal necrosis, and vascular congestion/hemorrhage, using the following grades: 0 = none, 1 = mild, 2 = moderate, and 3 = severe. Three non-overlapping fields per slide were assessed at ×400 magnification, and mean scores were calculated for each experimental group.

### Statistical analysis

All data were expressed as mean ± standard deviation (SD). Statistical analyses were performed using GraphPad Prism version 8.4 (GraphPad Software, San Diego, CA, United States). Data normality was verified using the Shapiro–Wilk test. For most biochemical and histological parameters, one-way analysis of variance (ANOVA) followed by Tukey’s *post hoc* test was applied to compare differences among the groups. For body weight measurements, which involved repeated weekly assessments across time, two-way ANOVA (group × time) followed by Tukey’s multiple comparisons test was used. Differences were considered statistically significant at p < 0.05.

## Results

### Allicin, metformin, and their combination as modulators of body and relative heart weights in doxorubicin-induced cardiotoxicity in rats

**FIGURE 1:**
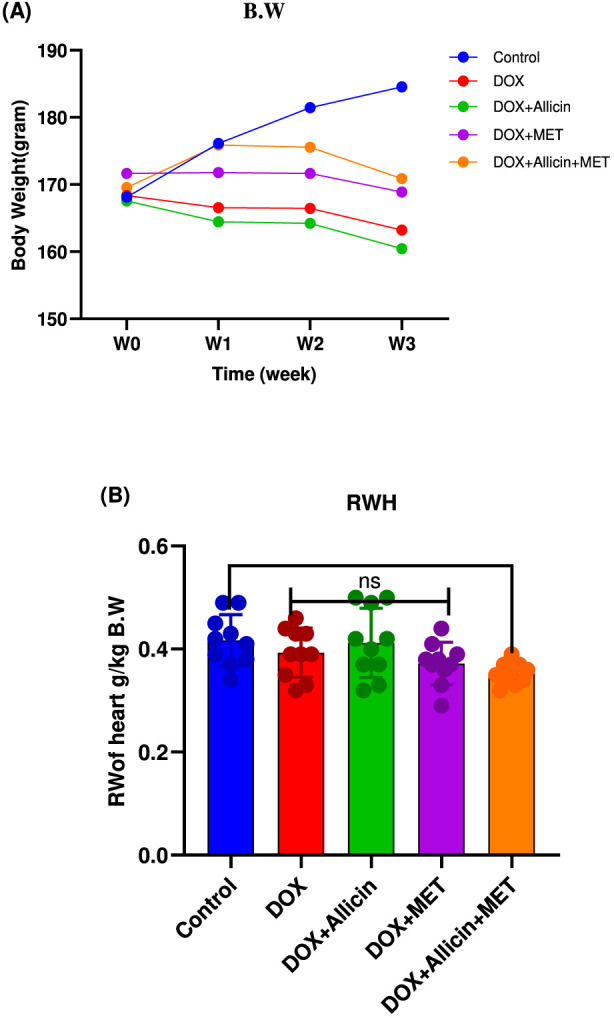
Allicin, metformin, and their combination as modulators of body and relative heart weights in doxorubicin-induced cardiotoxicity in rats statistical analysis for body weight **(A)** was performed using two-way repeated-measures ANOVA (time × treatment) followed by Tukey’s *post hoc* test. Relative heart weight **(B)** was analyzed using one-way ANOVA followed by Tukey’s *post hoc* test. Data are presented as mean ± SD (n = 10). *p < 0.05 vs. control group.

Over the 3-week experimental period, no significant differences in body weight were detected among the study groups (p > 0.05; Figure 1). Similarly, relative heart weight showed no significant variation between the DOX and control groups (p = 0.7930). Comparable findings were observed in the DOX + Allicin and DOX + Metformin groups (p > 0.05 for all). However, the combined DOX + Allicin + Metformin treatment resulted in a significant reduction in relative heart weight compared with control (p = 0.0340).

### Allicin, metformin, and their combination attenuate cardiac enzyme alterations in a rat model of doxorubicin-induced cardiotoxicity

**FIGURE 2:**
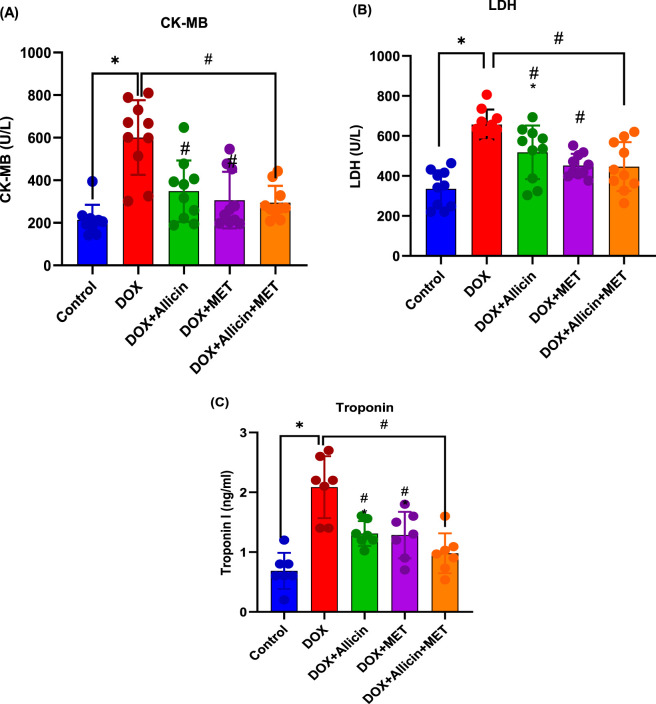
Allicin, metformin, and their combination attenuate cardiac enzyme alterations in a rat model of doxorubicin-induced cardiotoxicity. **(A)** Creatine kinase-MB (CK-MB), **(B)** lactate dehydrogenase (LDH), and **(C)** cardiac troponin I (cTn-I). Data are presented as mean ± SD (n = 10). Statistical analysis was performed using one-way ANOVA followed by Tukey’s *post hoc* test. *p < 0.05 vs. control group, #p < 0.05 vs. DOX group.

Serum cardiac injury biomarkers (CK-MB, LDH, and cTn-I) were markedly elevated in the DOX group compared with the control (p < 0.0001), confirming substantial myocardial damage (Figure 2). Co-administration of allicin with DOX significantly reduced these elevations (p < 0.05 vs. DOX for all markers), although LDH, and cTn-I levels remained higher than control values (p < 0.05 vs. control). Metformin treatment also attenuated DOX-induced elevations in cardiac enzymes. LDH and CK-MB levels were restored to values not significantly different from the control (p > 0.05), whereas cTn-I remained mildly elevated (p = 0.0333 vs. control). Importantly, the combined allicin–metformin therapy produced the greatest improvement across all biomarkers. CK-MB and LDH levels were fully normalized (p > 0.05 vs. control; p < 0.0001 vs. DOX), and cTn-I was substantially reduced to near-control levels with no significant difference from the control group (p = 0.5644).

### Ameliorative role of allicin, metformin, and their combination on antioxidant biomarkers in doxorubicin-induced cardiotoxicity in rats

**FIGURE 3:**
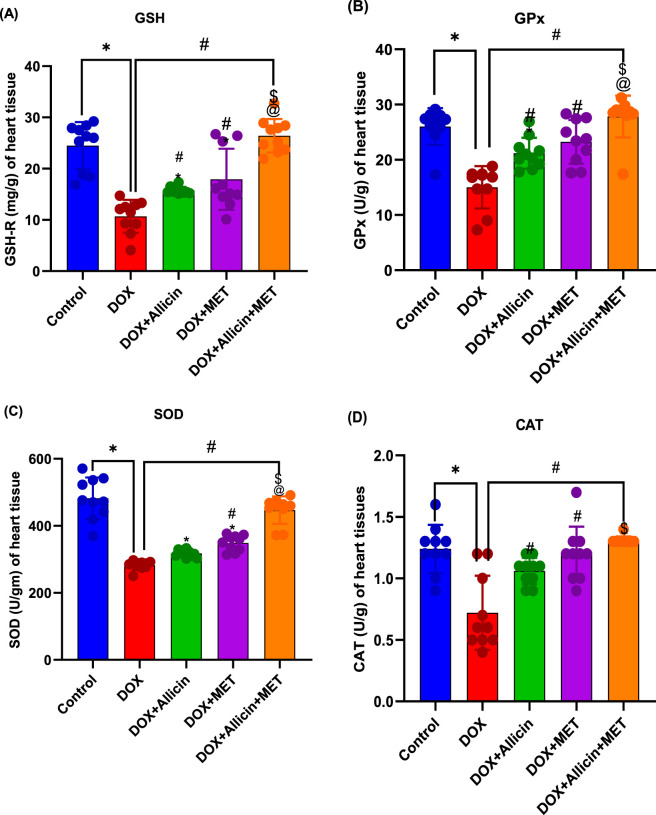
Ameliorative role of allicin, metformin, and their combination on antioxidant biomarkers in doxorubicin-induced cardiotoxicity in rats. **(A)** Glutathione reductase (GSH), **(B)** Glutathione peroxidase (GPx), **(C)** Superoxidase dismutase, **(D)** Catalase (CAT). Data are presented as mean ± SD (n = 10). Statistical analysis was performed using one-way ANOVA followed by Tukey’s *post hoc* test. *p < 0.05 vs. control group, #p < 0.05 vs. DOX group, $p < 0.05 vs. DOX + Allicin group, @p < 0.05 vs. DOX + MET group.

Antioxidant enzyme activities (GSH-R, GPx, SOD, and CAT) were significantly reduced in the DOX group compared with the control (p < 0.0001 for all; Figure 3), indicating marked oxidative stress. Treatment with either allicin or metformin partially restored these antioxidant defenses (p < 0.05 vs. DOX), though levels remained below control values. Notably, the DOX + Allicin + Metformin combination restored antioxidant enzyme activities to control levels (p > 0.05) with significant improvement over the DOX and single-treatment groups (p < 0.05).

### Allicin, metformin, and their combination reduce lipid peroxidation (MDA levels) in a rat model of doxorubicin-induced cardiotoxicity

**FIGURE 4:**
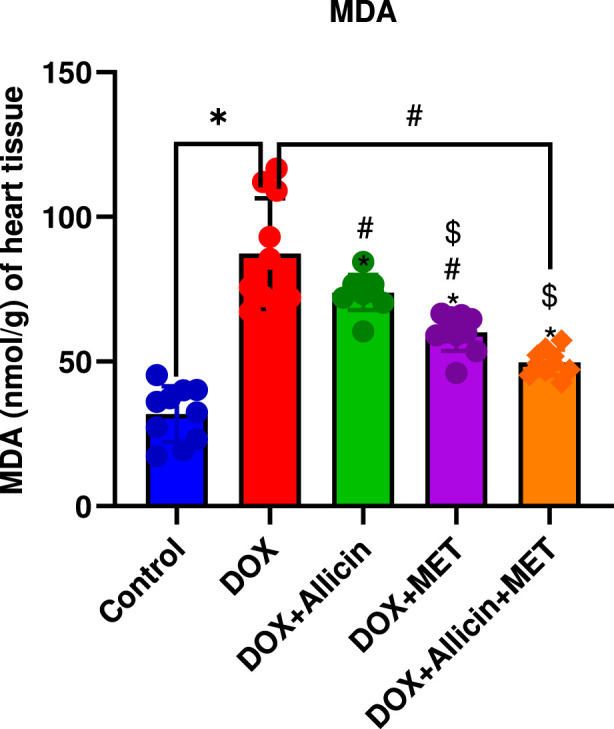
Allicin, metformin, and their combination reduce lipid peroxidation (MDA levels) in a rat model of doxorubicin-induced cardiotoxicity. Data are presented as mean ± SD (n = 10). Statistical analysis was performed using one-way ANOVA followed by Tukey’s *post hoc* test. *p < 0.05 vs. control group, #p < 0.05 vs. DOX group, $p < 0.05 vs. DOX + Allicin group.

Malondialdehyde (MDA) levels were markedly elevated in the DOX group relative to control (p < 0.0001; Figure 4), confirming increased lipid peroxidation and oxidative damage. Both allicin and metformin treatments significantly decreased MDA levels compared with DOX (p < 0.05), although they remained above control. The combined therapy produced a more pronounced reduction (p < 0.0001 vs. DOX), yet values were still significantly higher than control (p = 0.0040).

### Allicin, metformin, and their combination restore nitric oxide levels in a rat model of doxorubicin-induced cardiotoxicity

**FIGURE 5:**
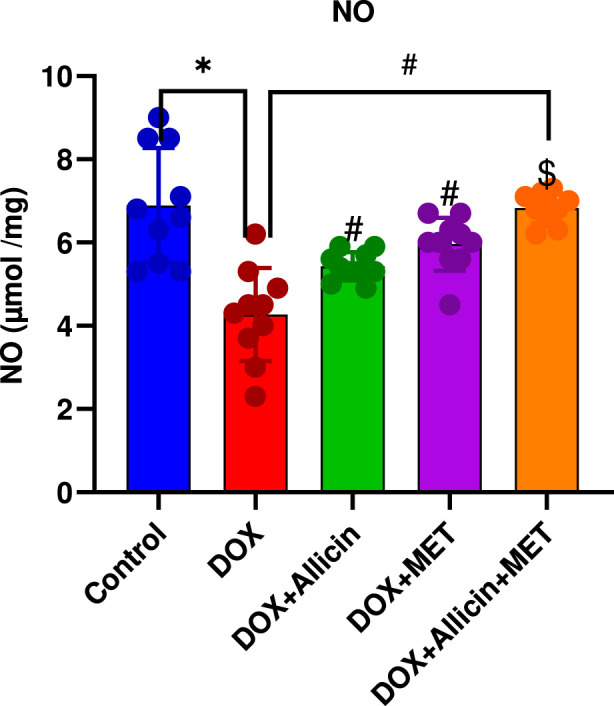
Allicin, metformin, and their combination restore nitric oxide levels in a rat model of doxorubicin-induced cardiotoxicity. Data are presented as mean ± SD (n = 10). Statistical analysis was performed using one-way ANOVA followed by Tukey’s *post hoc* test. *p < 0.05 vs. control group, #p < 0.05 vs. DOX group, $ p < 0.05 vs. DOX+ Allicin group.

As shown in Figure 5, NO levels were significantly reduced in the DOX group compared with the control (p < 0.0001). Treatment with either allicin or metformin partially restored NO levels (p < 0.05 vs. DOX), although they remained below control values. In contrast, the DOX + Allicin + Metformin combination completely normalized NO expression (p > 0.05 vs. control; p < 0.0001 vs. DOX), indicating improved endothelial function and nitric oxide bioavailability under combined therapy.

### Histopathological evaluation

Histopathological examination revealed normal myocardial architecture in the control group, with well-organized fibers and absence of cellular infiltration or necrosis. In contrast, the DOX group exhibited severe myocardial injury characterized by myofibrillar degeneration, cytoplasmic vacuolization, necrosis, and marked inflammatory cell infiltration.

Treatment with allicin produced partial structural recovery, reflected by reduced inflammatory infiltration and improved fiber alignment, although some degenerative changes remained. Metformin treatment resulted in more pronounced improvement, with fewer necrotic cells and better-preserved myocardial structure.

**FIGURE 6:**
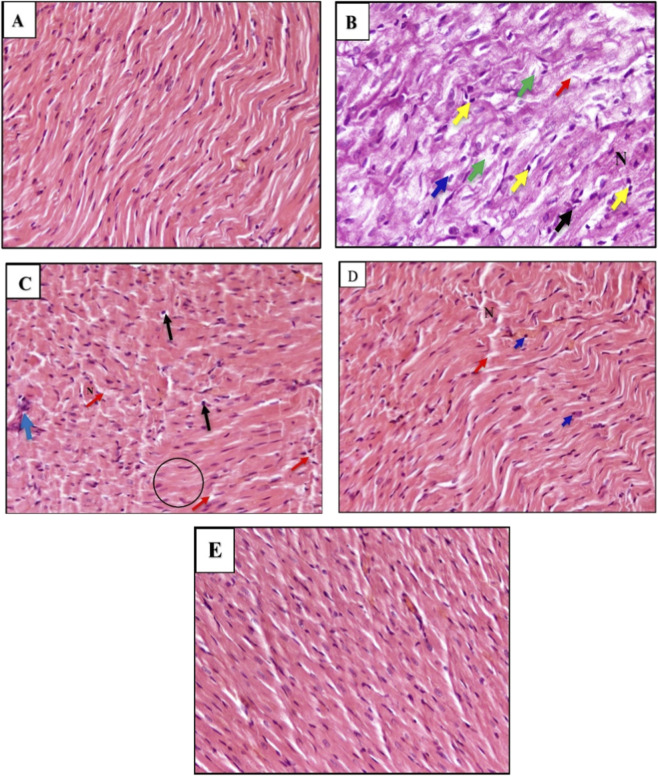
Histological photomicrographs of cardio sections of tissue from experimental rats were stained with Hematoxylin and Eosin (H&E), showing: **(A)** Normal Control Group showed: The myocardial architecture was well-preserved, with regular, parallel alignment of cardiac muscle fibers. There were no signs of cellular infiltration, necrosis, or interstitial edema [400×] Scale bar (50 µm). **(B)** Doxorubicin (DOX)-Treated Group showed: pathological alterations. These included widespread degeneration (Green arrow); a moderate nuclear pyknosis (Yellow arrows); inflammatory cell infiltration (Black arrows); cytoplasmic vacuolization (Blue arrow) and necrosis cells (N) and moderate of cardiac fibers fragmentation (Red arrow); were observed [400×] [100×] Scale bars (50 µm). **(C)** Doxorubicin (DOX) + Allicin Group showed: moderate histological improvement compared to the DOX-only group. Partial restoration of fiber alignment and some structural alterations persisted, such as a few scattered inflammatory cells (Blue arrow) were observed; pyknotic nuclei (Black arrows); necrosis cells (N); and loss of myocardial cellular constituents (Black circle) [400×] Scale bars B= (50 µm). **(D)** Doxorubicin (DOX) + Metformin Group showed: reduction in inflammatory cell infiltration; a few pyknotic nuclei (blue arrows) mild necrotic cells (N) and a few muscle fibers having fragmentation (red arrow); were observed [400×] Scale bars B= (50 µm). **(E)** DOX + Allicin +Metformin Group showed: Cardiac muscle fibers were nearly restored to normal morphology, with well-organized structure, minimal vacuolization, and absence of inflammatory infiltration or necrosis [400×] Scale bars B= (50 µm).

Notably, the DOX + Allicin + Metformin group demonstrated the greatest histological restoration, showing myocardial fibers that were nearly comparable to the control architecture, with minimal vacuolization and markedly reduced inflammatory and necrotic changes (Figure 6).

These morphological findings were further supported by the semi-quantitative histopathological scoring (Table 1), which showed that the DOX + Allicin + Metformin group achieved the lowest overall injury score among all DOX-treated groups, confirming its superior structural improvement.

**TABLE 1:** Histopathological scoring of cardiac tissue.

Group	Degeneration	Pyknosis	Cell infiltration	Necrosis cells	Fibers fragmentation	Total score
Control	0	0	0	0	0	0
DOX	2	2	2	2	2	10
DOX +Allicin	2	2	1	1	1	7
DOX +Metformin	1	1	1	1	1	5
DOX+ Allicin+ Metformin	0	1	1	0	0	2

The slides were re-examined and evaluated using a microscope (Olympus, BX53) and scored semi-quantitative scoring system (0, no pathology observed; 1, mild; 2, moderate; 3, severe) for Myofiber integrity, Inflammation, Vacuolization, Necrosis and Congestion/Hemorrhage.

## Discussion

Doxorubicin (DOX) remains an essential chemotherapeutic agent; however, its dose-dependent cardiotoxicity continues to limit its clinical utility. The present study investigated the potential cardioprotective of metformin and allicin, individually and in combination, against doxorubicin (DOX)-induced cardiotoxicity in male albino rats.

The administered DOX regimen (18 mg/kg cumulative dose) successfully induced consistent biochemical and histopathological hallmarks of cardiotoxicity, characterized by a significant rise in serum CK-MB, LDH, and troponin I levels—clear indicators of cardiomyocyte membrane damage and leakage of intracellular enzymes. These biochemical disruptions were accompanied by a marked depletion of endogenous antioxidants (GSH, GPx, SOD, and CAT) and an elevation of the lipid peroxidation marker MDA, demonstrating a profound redox imbalance. Concomitant reduction in NO levels further reflected endothelial dysfunction, likely resulting from eNOS uncoupling and increased superoxide anion generation. The histopathological findings of myofibrillar loss, cytoplasmic vacuolation, interstitial edema, and inflammatory infiltration closely parallel earlier descriptions of DOX-induced myocardial degeneration (Songbo et al., 2019; Zhang et al., 2023). The slight, non-significant reduction in relative heart weight also aligned with previously reported cardiac atrophic changes (Abdelrahman, 2020). This absence of a statistically significant change in heart weight is not unexpected, as early or mild DOX-induced atrophic responses may occur without producing detectable differences in gross organ mass. Importantly, this does not contradict the clear biochemical and histological evidence of cardiotoxicity observed in the present model, which is consistent with prior studies showing that molecular and structural injury often precede measurable alterations in cardiac weight. Collectively, these findings validate the robustness of the experimental model and reaffirm the centrality of oxidative and inflammatory mechanisms in DOX cardiotoxicity.

Metformin treatment (300 mg/kg/day, p.o.) exerted marked restorative effects on all measured parameters. The significant reductions in CK-MB, LDH, cTn-I, and MDA levels, together with the enhancement of antioxidant defenses (GSH, GPx, SOD, and CAT), indicate effective attenuation of DOX-induced oxidative injury. The concurrent normalization of NO levels further suggests preserved endothelial function.

Although the present study did not directly quantify AMPK activation or apoptotic and inflammatory signaling, the pattern of biochemical recovery observed here is highly consistent with the mechanisms reported in earlier studies. Previous investigations demonstrated that metformin activates AMP-activated protein kinase (AMPK), leading to improved mitochondrial function, upregulation of antioxidant genes, suppression of ROS generation, and inhibition of mitochondrial apoptotic pathway (Dihoum et al., 2023; Ikewuchi et al., 2021; Kamel et al., 2022; Maghraby et al., 2025) These established mechanisms provide a plausible explanation for the protective profile detected in our study.

Histologically, myocardial sections from metformin-treated rats exhibited better preservation of myofibrillar architecture with minimal inflammatory infiltration compared to the DOX group, which aligns with metformin’s well-documented antioxidative, anti-inflammatory, and anti-apoptotic actions described in the literature.

Similarly, allicin (40 mg/kg/day) demonstrated substantial cardioprotective effects. In our study, allicin treatment significantly attenuated cardiac enzyme leakage and reduced MDA levels while restoring key antioxidant biomarkers and improving NO bioavailability. These biochemical improvements align with allicin’s well-established redox-modulating properties. Sulfur-containing constituents of allicin possess potent direct ROS-scavenging activity, which has been extensively documented in previous research. In addition, earlier studies have shown that allicin can modulate major pro-oxidant and pro-inflammatory pathways—particularly NADPH oxidase and NF-κB signaling—thereby limiting oxidative and inflammatory injury (Aboubakr et al., 2023; Samra et al., 2020). Although our study did not directly quantify these molecular pathways, the observed improvements in antioxidant status and NO levels are consistent with these reported mechanisms. Histologically, allicin-treated myocardial tissue showed meaningful structural recovery, albeit slightly less pronounced than that observed with metformin, yet still in agreement with previous reports demonstrating attenuation of DOX-induced myocardial degeneration following allicin administration (Gao et al., 2024).

The metformin + allicin combination produced the most pronounced protection against DOX-induced cardiotoxicity. This group exhibited the greatest normalization of serum cardiac injury biomarkers (CK-MB, LDH, and troponin-I), near-complete restoration of endogenous antioxidant defenses (GSH, GPx, SOD, CAT), and a marked decline in lipid peroxidation (MDA). NO bioavailability was also effectively preserved. These biochemical findings were strongly supported by histopathological examination, which showed myocardial fibers and architecture closely resembling the control group, with minimal residual injury.

A mechanistic basis for the improved effect observed with the combination of metformin and allicin can be explained by their modulation of complementary pathways involved in doxorubicin induced cardiotoxicity. Metformin activates the AMPK signaling pathway, which enhances mitochondrial function, limits mitochondrial ROS production, and promotes antioxidant defense through downstream stimulation of Nrf2-dependent cytoprotective genes.

Allicin, in contrast, targets pathways responsible for acute oxidative and inflammatory injury by inhibiting NF-κB activation, suppressing NADPH oxidase–derived ROS, and supporting endothelial nitric oxide production via modulation of the eNOS/NO pathway. Given that doxorubicin-induced cardiac injury is driven by mitochondrial dysfunction, excessive ROS, impaired NO signaling, and NF-κB–mediated inflammation, the convergence of AMPK–Nrf2 activation (metformin) with NF-κB/NOX inhibition and NO restoration (allicin) provides a mechanistically consistent explanation for the enhanced cardioprotective profile observed with the combined therapy.

This interaction offers a plausible explanation for the near-normalization of oxidative stress markers and cardiac biomarkers observed in the combination group. While the present findings clearly demonstrate the superior cardioprotective effect of the metformin–allicin combination, additional work is needed to elucidate the underlying mechanisms in greater depth. Future studies should incorporate formal quantitative synergy analyses (e.g., isobolographic or combination-index methods) to determine whether the enhanced efficacy represents true pharmacodynamic synergy. Moreover, extended functional assessments such as echocardiography, together with quantitative molecular analyses, would help clarify downstream pathways contributing to the observed protection. Clinical investigations will ultimately be required to evaluate the translational potential of this combination.

In conclusion, Metformin and allicin each conferred significant cardioprotection against doxorubicin-induced cardiotoxicity, evidenced by the restoration of cardiac enzymes, reduction of oxidative stress, and improvement in myocardial histoarchitecture. Notably, combined therapy produced greater biochemical and structural recovery than either monotherapy, highlighting its enhanced overall cardioprotective efficacy.

## Data Availability

The original contributions presented in the study are included in the article/Supplementary Material, further inquiries can be directed to the corresponding author.
