# Supplementary material for: Dual therapy with allicin and metformin provides superior cardioprotection against doxorubicin-induced cardiotoxicity in rats compared to monotherapy
Source: Front Pharmacol. 2026 Jan 9;16:1725943. doi: 10.3389/fphar.2025.1725943 (PMC12827077; doi:10.3389/fphar.2025.1725943)
Supplement: Supplementary file 1 [file DataSheet1.PDF]

Supplementary Table 1: Relative heart weight, Biochemical and oxidative stress markers (One-way ANOVA)

Parameter	control	DOX	DOX+ Allicin	DOX+MET	DOX+ Allicin+ MET
**RWH g/kg B. W**	**0.42 ± 0.05**	**0.39 ± 0.05**	**0.41 ± 0.07**	**0.37 ± 0.04**	**0.36 ± 0.02**
**CK-MB (U/L)**	**214.0± 70**	**600.8± 175 ***	**348.5±144 #**	**306.6± 132 #**	**294.5 ±79 #**
**LDH (U/L)**	**335.1± 92**	**657.2 ± 74.7 ***	**518.0 ±134* #**	**452.4± 57 #**	**446.5±122 #**
**C T n-I (ng/mL)**	**0.68± 0.30**	**2.08 ± 0.51 ***	**1.3 ± 0.20 * #**	**1.28± 0.38* #**	**0.98 ±0.33 #**
**GSH (mg/g)**	**24.5 ± 4.61**	**10.7 ± 3.21** *	**15.8 ± 0.69 *** $^{\#}$	**17.9 ± 6.0 *** $^{\#}$	**26.4 ±3.26** $^{\# \$ @}$
**GPX (U/g)**	**26.0 ± 3.34**	**15.0 ± 3.8** *	**21.2 ± 2.8**$^{\#}$	**23.2 ± 3.9** $^{\#}$	**27.8 ±3.8** $^{\# \$ @}$
**SOD (U/g)**	**482 ± 62**	**282 ± 13** *	**317 ± 11***	**348 ± 23**$^{* \#}$	**446 ± 40.9** $^{\# \$ @}$
**CAT (U/g)**	**1.24 ± 0.19**	**0.72 ± 0.30 ***	**1.06 ± 0.10 #**	**1.22 ± 0.22 #**	**1.3 ±0.2 #**
**MDA (nmol/g)**	**31.8 ± 9.5**	**87.3 ± 19.1 ***	**73.8 ±6.0 *** $^{\#}$	**60.0 ± 6.3*** $^{\# \$}$	**49.6 ±4.4*** $^{\# \$}$
**NO× (µmol/L)**	**6.89 ± 1.37**	**4.27 ± 1.11** *	**5.43 ± 0.34** $^{* \#}$	**5.96 ± 0.63** $^{\#}$	**6.83 ± 0.36**$^{\$ \#}$

Data are presented as mean ±SD (n = 10). Statistical analysis was performed using one-way ANOVA followed by Tukey's post hoc test. * $p < 0.05$ vs control group, # $p < 0.05$ vs DOX group, $ $p < 0.05$ vs DOX + Allicin group, @ $p < 0.05$ vs DOX + MET group.

Supplementary Table 2: Body weight at different time points (Two-way ANOVA)

group	W0	W1	W2	W3
**control**	**168.1± 15**	**176.1± 16**	**181.4±16**	**184.5±17**
**DOX**	**168.3± 18**	**166.5± 18**	**166.4± 19**	**163.2±19**
**DOX+ Allicin**	**167.5±17**	**164.4±18**	**164.2±18**	**160.5±18**
**DOX+ Met**	**171.6± 19**	**171.7 ±22**	**171.6 ± 20**	**168.8±19**
**DOX + Allicin+ Met**	**169.5±14**	**175.8±17**	**175.5±17**	**170.8±16**

Data are presented as mean ±SD (n = 10). Statistical analysis was performed using Two-way ANOVA
